# The prognostic value and potential mechanism of Matrix Metalloproteinases among Prostate Cancer

**DOI:** 10.7150/ijms.46780

**Published:** 2020-06-21

**Authors:** Xinyu Geng, Chunyang Chen, Yuhua Huang, Jianquan Hou

**Affiliations:** Department of Urology, The First Affiliated Hospital of Soochow University, 215006, Suzhou, Jiangsu, China.

**Keywords:** prostate cancer, matrix metalloproteinases, prognostic signature, LASSO regressio

## Abstract

**Background:** Matrix Metalloproteinases (MMPs) play an indispensable role in the initial alteration and development of PCa. We tried to generate an MMP-related prognostic signature (MMPS) in prostate cancer (PCa).

**Methods:** TCGA-PRAD, MSKCC/GSE21032, GSE116918, GSE70769 cohorts were enrolled to assess the prognostic value of MMPs. The least absolute shrinkage and selection operator (LASSO) Cox regression was employed to generate the MMPS signature. The log-rank test and Kaplan-Meier (K-M) survival curve were applied to show the difference RFS, The receiver operating characteristic (ROC) curve and area under the ROC curve (AUC) was plotted to predict the accuracy of signature. CIBERSORT was conducted to analyze the different immune infiltration in MMPS-H and MMPS-L groups. Potential signaling pathways activated in the MMPS-H groups by Metascape.

**Results:** MMP1, MMP7, MMP11, MMP24 and MMP26 were selected by LASSO regression and established the MMPS predict signature. The MMPS showed the high prognostic value in TCGA-PRAD training cohort (AUC=0.714) and validation cohorts (GSE116918: AUC=0.976, GSE70769: AUC=0.738, MSKCC: AUC=0.793). Pid integrin1 pathway, G2M checkpoint, and response to growth factor signaling pathways were activated in MMPS-H group, patients with the high MMPS risk score and low M2 macrophage showed the worst recurrence-free survival (RFS).

**Conclusion:** MMPs involved and played an essential role in the tumorigenesis and biochemical recurrence in PCa patients. The MMPS signature could accurately predict the recurrence of PCa patients and validated in several cohorts.

## Introduction

Prostate cancer (PCa) caused a massive health burden for males, especially older males, around the whole world. The cancer-specific mortality of PCa is high to 6.6%, there are more than three hundred thousand patients die from PCa each year 1-3. Methods to inhibit the function of androgen receptor are sufficient to protect the PCa patients; however, the effeteness could only insist for about two years, and finally step into the advanced castration-resistant PCa (CRPC) 4-7. Therefore, it is necessary to find the prognostic signature and potential treatment targets to protect the patients from the harm of the advanced PCa stage.

Matrix Metalloproteinases (MMPs) are zinc-dependent proteases that display a specific proteolytic activity against a broad range of substrates located on the extracellular matrix (ECM). There are about 23 MMPs were reported in human beings to regulate the cell functions through growth factors, cell-surface receptors, cytokines, chemokines and proteases 8, 9. The typical structure of MMPs consists of an N-terminal zymogenic propeptide domain (~80 amino acids), a metal-dependent catalytic domain (~170 amino acids), a linker region (~15-65 amino acids), and a C-terminal hemopexin-like domain (~200 amino acids) 10, 11. The relevant physiological function of MMPs, as well as the increased or decreased expression of them could affect the development of diabetes, neurological disorders, chronic inflammatory disease and cancers 12-16. The MMPs are divide in different based on the function, MMP1, MMP8 and MMP13 belong to collagenases, MMP7 and MMP26 are matrilysins, MMP3, MMP10 and MMP11 are stromelysins, MMP2 and MMP9 are gelatinases, MMP14-17, MMP24 and MMP25 are in the membrane-type group, MMP 12 is metalloelastase, MMP20 is enamelysin, while the other MMPs are not in any specific subgroup 17.

In the past decades, several pieces of research demonstrated the function of MMPs in cancer. Qi *et al*. 18 reported that the CCL7 chemokine could regulate the MMP-9 mediated collagen degradation to promote the invasion and metastasis of liver cancer. Furuya *et al*. 19 illustrated a urine-based protein-determined signature to predict the bladder diagnostic, of which including MMP9 and MMP10. Singh *et al*.^20^ revealed a 15 gene signature, identified by macrophages-tumor interactions, to predict the outcome of breast cancer, of which including MMP1 and MMP9. Therefore, we consider building a MMPs related prognostic signature in PCa patients.

## Methods

### Data acquirement from enrolled cohorts

In the current study, we concerned the TCGA-PRAD cohort with the RNA sequence data from the platform based on the Illumina system and clinical characteristics download from UCSC (https://gdc.xenahubs.net/download/TCGA-PRAD.htseq_fpkm.tsv.gz), We transferred the data from fragments per kilobase of non-overlapped exon per million fragments (FPKM) to into transcripts per kilobase million (TPM) values. Those mRNAs with TPM less than 1 in over 90% samples were considered noise and removed from downstream analysis. There are 52 normal samples and 500 tumor samples from 496 patients in TCGA-PRAD cohort. Meanwhile, sequence data of three cohorts from the Gene Expression Omnibus 36 (GEO, http://www.ncbi.nlm.nih.gov/geo/) was also obtained, including MSKCC/GSE21032, GSE116918, GSE70769 cohorts. The details of all the four cohorts are listed in Table 1. The genetic alterations of MMPs in patients with PCa were illustrated via the cBioPortal platform (http://www.cbioportal.org/) 21, 22, which recorded the missense and truncating mutations as well as amplification and deep deletion. The correlation between DNA methylation and mRNA expression of each MMP was also tested with cBioPortal.

### Establishment and validation of MMP-related prognostic signature

The least absolute shrinkage and selection operator (LASSO) Cox regression models were constructed using the package “glmnet”. By utilizing all the concerned genes, the function returns a series of values of λ and models. The coefficients of the majority of the original genes were penalized to zero in line with the increasing values of the tuning parameter λ. The λ was chosen when the partial likelihood deviance reached its lowest. A suitable model was chosen based on the 10-fold cross-validation of the function cv.glmnet. Using the function lambda.min, the remaining genes with non-zero LASSO coefficients were obtained. The risk score for each patient with LAC was calculated using the linear combination of each TPM of the gene multiplied by the LASSO coefficient of each gene. For the validation in three GEO cohorts, the risk score of each patient was also calculated by the MMP-related prognostic signature.

The log-rank test and Kaplan-Meier (K-M) survival curve were employed to show the difference RFS in both signal gene expression and risk score polarized high and low groups, while Cox regression conducted to generate the hazard ratio (HR) from high to low groups, with the use of “survival” and “survminer” packages. The receiver operating characteristic (ROC) curve and area under the ROC curve (AUC) was plotted to predict the accuracy of the signature.

### Different expression genes (DEGs) identification and pathway enrichment

The DEGs analysis was performed with R package “limma”, and the fold-change > 0.5 and P-value < 0.05 were used as criteria to screen for DEGs between the MMP- related high and low risk groups. We subsequently performed a functional enrichment analysis to find the potential signaling pathways activated in the high risk groups by Metascape (http://metascape.org) 23.

### Immune infiltration and prognostic value

To furthermore analysis how immune cell affects the PCa patients in the high and low MMP-related risk groups, as well as the combined prognostic value, we use the TPM data to generate the results of the infiltration rate of 22 immunocytes with CIBERSORT (https://cibersort.stanford.edu/) 24, in the estimated analysis, the Mixture file was made with gene symbol and sequencing values, one thousand was set as permutation value for statistical analysis, and disable quantile normalization was selected. What's more, the association between gene expression and immune infiltration was also analyzed by a website called tumor immune estimation resource (TIMER) (https://cistrome.shinyapps.io/timer) served for analysis of tumor-infiltrating immune cells 25.

### Statistical methods

The Student's test was used for statistical comparison among two subtypes. The ANOVA test was operated for analysis of more than two groups. Pearson correlation coefficient test was employed to assess the relationship between two factors, co-expression of two genes, or with the risk score. The statistical analysis of this research was implemented through R language (https://www.r-project.org/). P-value <0.05 was considered to be statistically significant.

## Results

### A comprehensive view of MMPs in PCa

**Figure 1:**
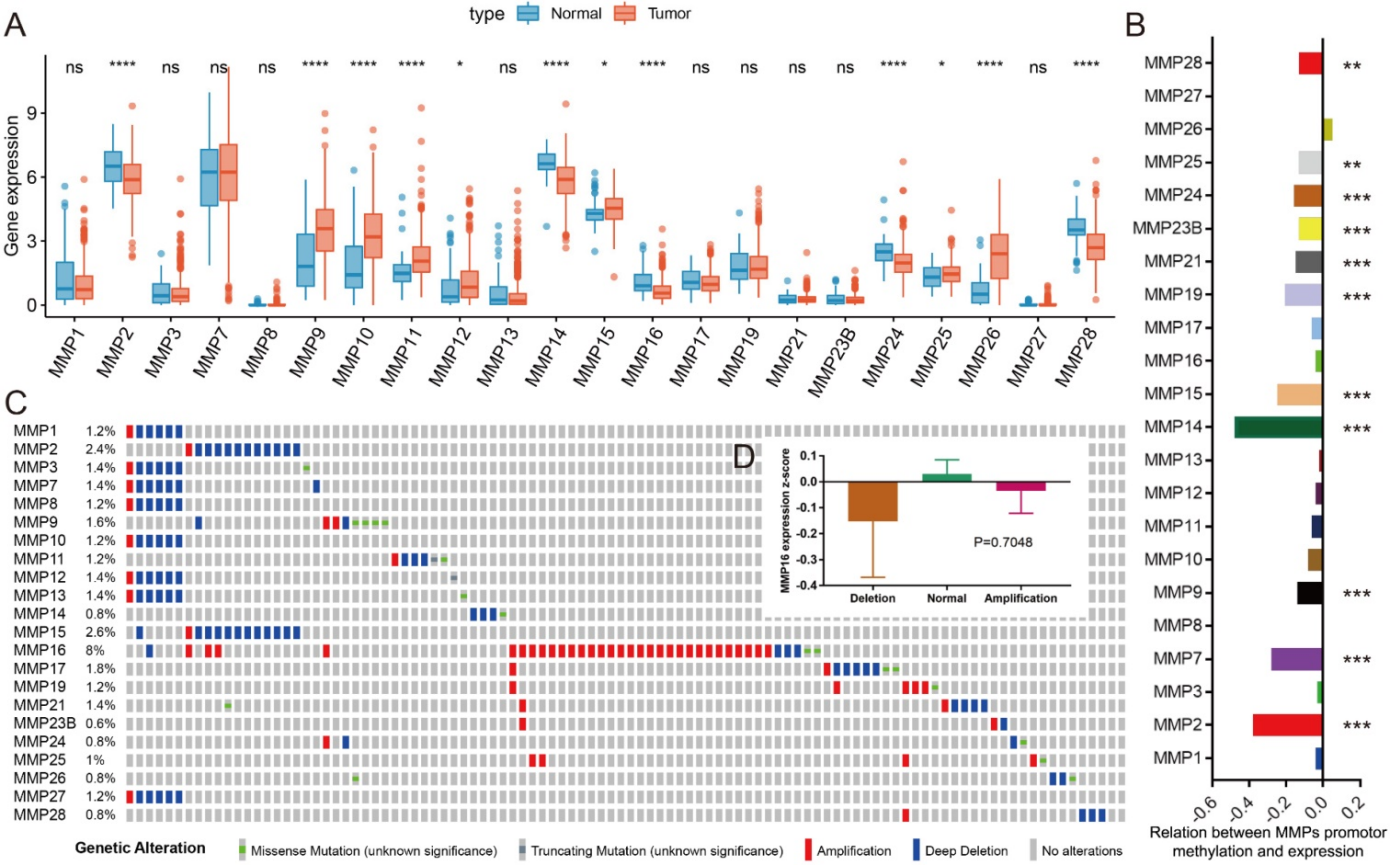
** Overview of MMPs in PCa.** (A) The mRNA expression of 22 MMPs among PCa tissues and normal prostate tissues; (B) The correlation of DNA methylation and mRNA expression of 22 MMPs; (C) The genetic alteration of 22 MMPs in PCa patients; (D) The amplification and deletion don't affect the mRNA expression of MMP16.

After the search of the literature and extract the available data from TCGA-PRAD, the expression of 22 MMPs were listed in** Figure 1A** comparing between normal and tumor samples. We could see that about half MMPs are highly expressed in tumor tissues as compared with normal tissues, including MMP9, MMP10, MMP11, MMP12, MMP15, MMP25, MMP26 (all, P<0.05), while some other MMPs decreased in tumor tissues, including MMP2, MMP14, MMP16, MMP24, MMP28(all, P<0.05). DNA methylation is one of the risk factors that impact the mRNA level of genes, we analyzed the association between DNA methylation and mRNA expression for MMPs, and found that the DNA methylation level of MMP2, MMP7, MMP9, MMP14, MMP15, MMP19, MMP21, MMP23B, MMP24, MMP25 and MMP28 could impact its mRNA level in PCa (all, P<0.05) (**Figure 1B**). Genetic alteration is another risk factor that affects mRNA expression. We found that the rate of genetic change in most MMPs was shallow in TCGA-PRAD patients, while the genetic alteration rate of MMP16 is high to 2.6% (**Figure 1C**), however, when we compared the mRNA expression of MMP16 in different genetic alteration subgroups, there was no difference, of which means that the genetic alteration of MMP16 does no effect to the mRNA expression (**Figure 1D**).

### Establishment of the MMP-related prognostic signature

**Figure 2:**
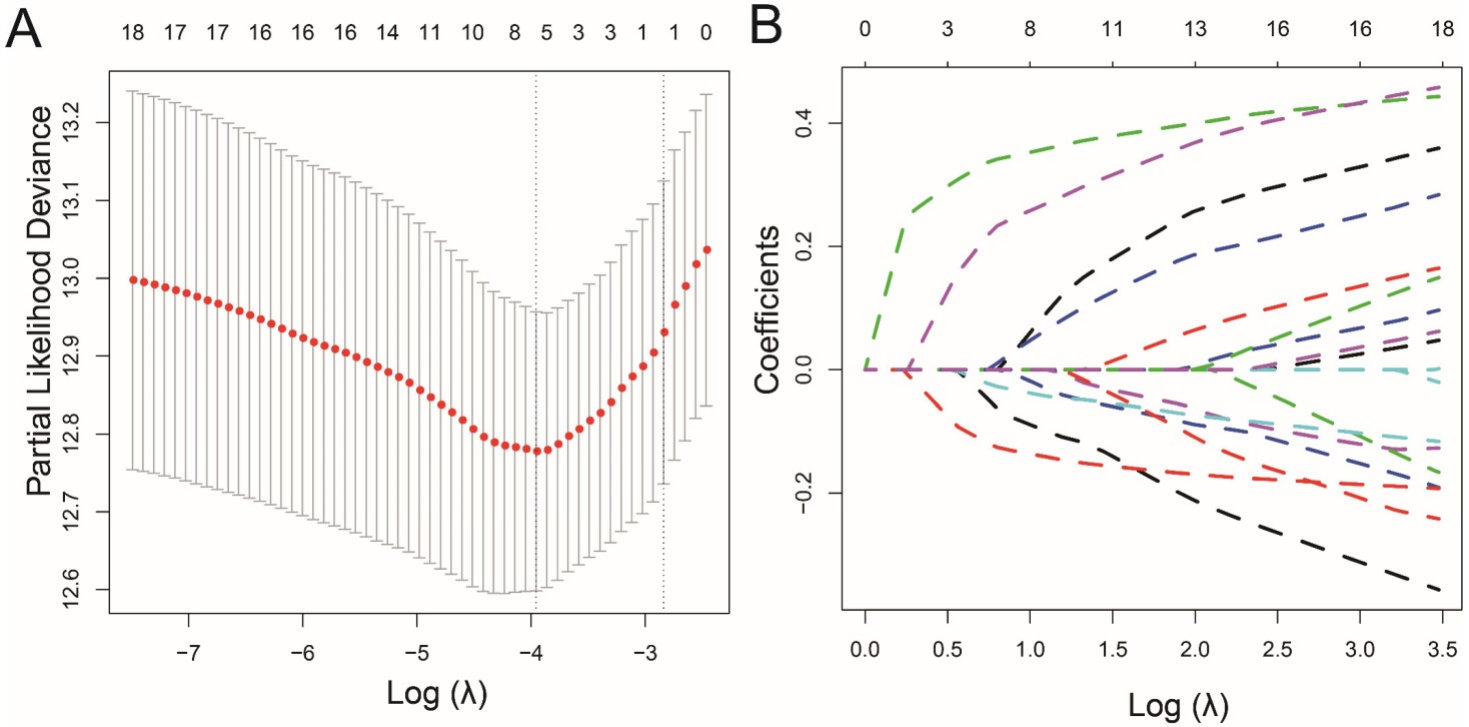
** LASSO analysis to screen the candidates for prognostic signature.** (A) The optimal tuning parameter (Lambda) in the LASSO model selected with the 10-fold cross-validation and one standard error rule; (B) LASSO coefficient profiles of the 18 MMPs.

**Figure 3:**
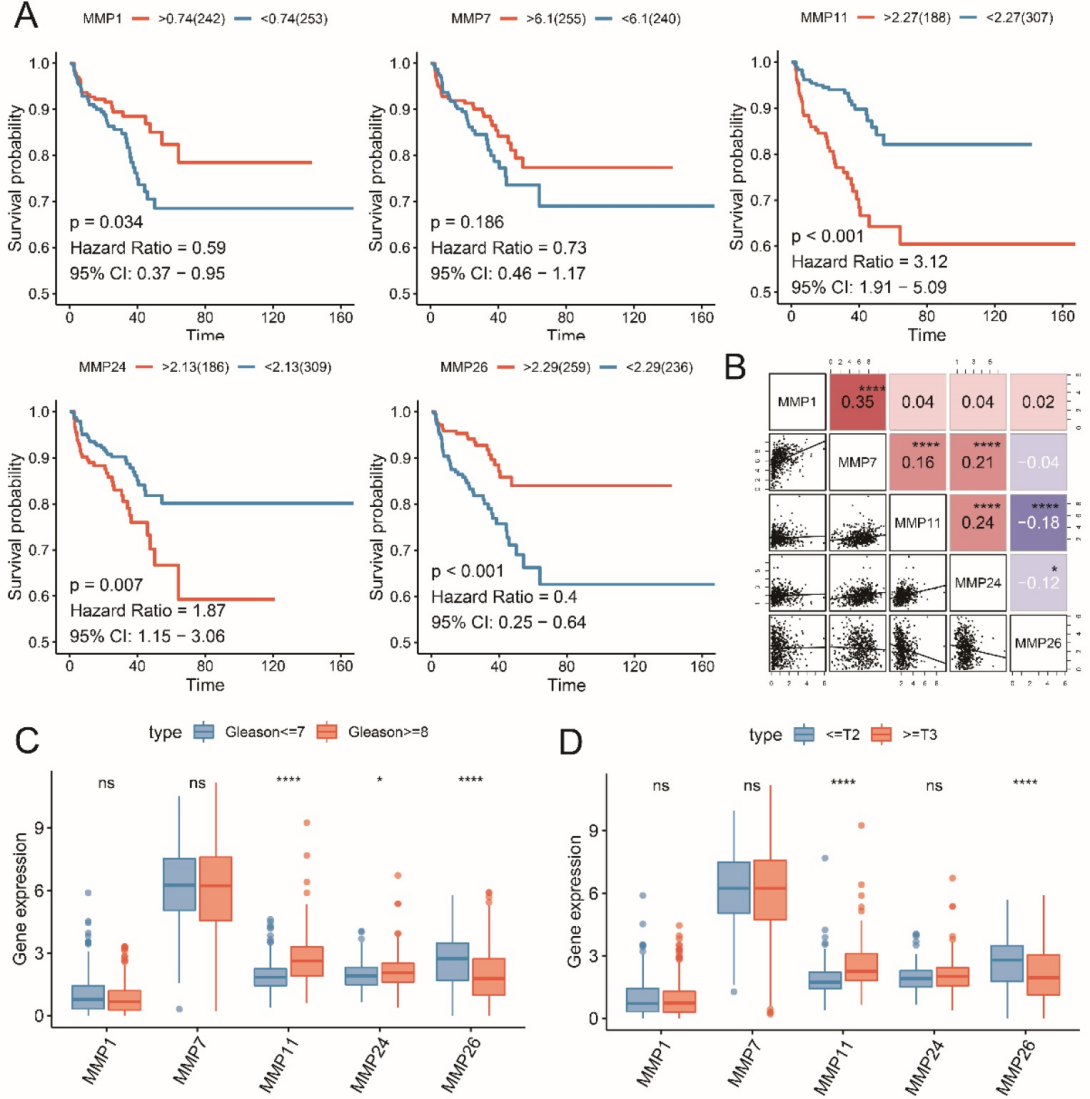
** Five MMPs candidates associated with the RFS and pathological stage.** (A) K-M plot showed the prognostic value of high and low level of five MMPs; (B) The correlation of mRNA expression of five MMPs; (C) The five MMPs expression in low and high Gleason score; (D) The five MMPs expression in early and advanced T stage.

We conducted the LASSO regression analysis to generate the signature, MMP8, MMP21, MMP23B and MMP27 were excluded by the low mRNA level, then 5 RFS associated MMPs were extracted to contract the prognostic signature, including MMP1, MMP7, MMP11, MMP24 and MMP26 (**Figure 2**). Then we reviewed the expression and prognostic value of these 5 MMPs. As shown in **Figure 3A**, MMP11 and MMP24 is the risk factors for unfavorable RFS (MMP11: HR=3.12, 95% CI=1.91-5.09, P<0.001; MMP24: HR=1.87, 95% CI=1.15-3.06, P=0.007), while MMP1 (HR=0.59, 95% CI=0.37-0.95, P=0.034), MMP7 (HR=0.73, 95% CI=0.46-1.17, P=0.186) and MMP26 (HR=0.40, 95% CI=0.25-0.64, P<0.001) are the protect factors for PCa patients and associated with the prolonged RFS Subsequently, we analyzed the correlation of the five MMPs expression and the expression form in different clinicopathological subgroups. MMP7 expression is highly associated with MMP1, MMP11 and MMP24, as well as MMP11 and MMP24, while MMP11 expression is negatively associated with MMP26 (**Figure 3B**, all, P<0.05). As to the expression in different Gleason score, MMP11 and MMP24 mRNA level is higher in advanced Gleason score ≥8 group, while MMP26 is higher in Gleason score ≤7 group (**Figure 3C**, all, P<0.05). To pathology T stage subgroups, patients with the advanced ≥ T3 stage shown a higher MMP11 and lower MMP26 (**Figure 3D**, all, P<0.05).

**Figure 4:**
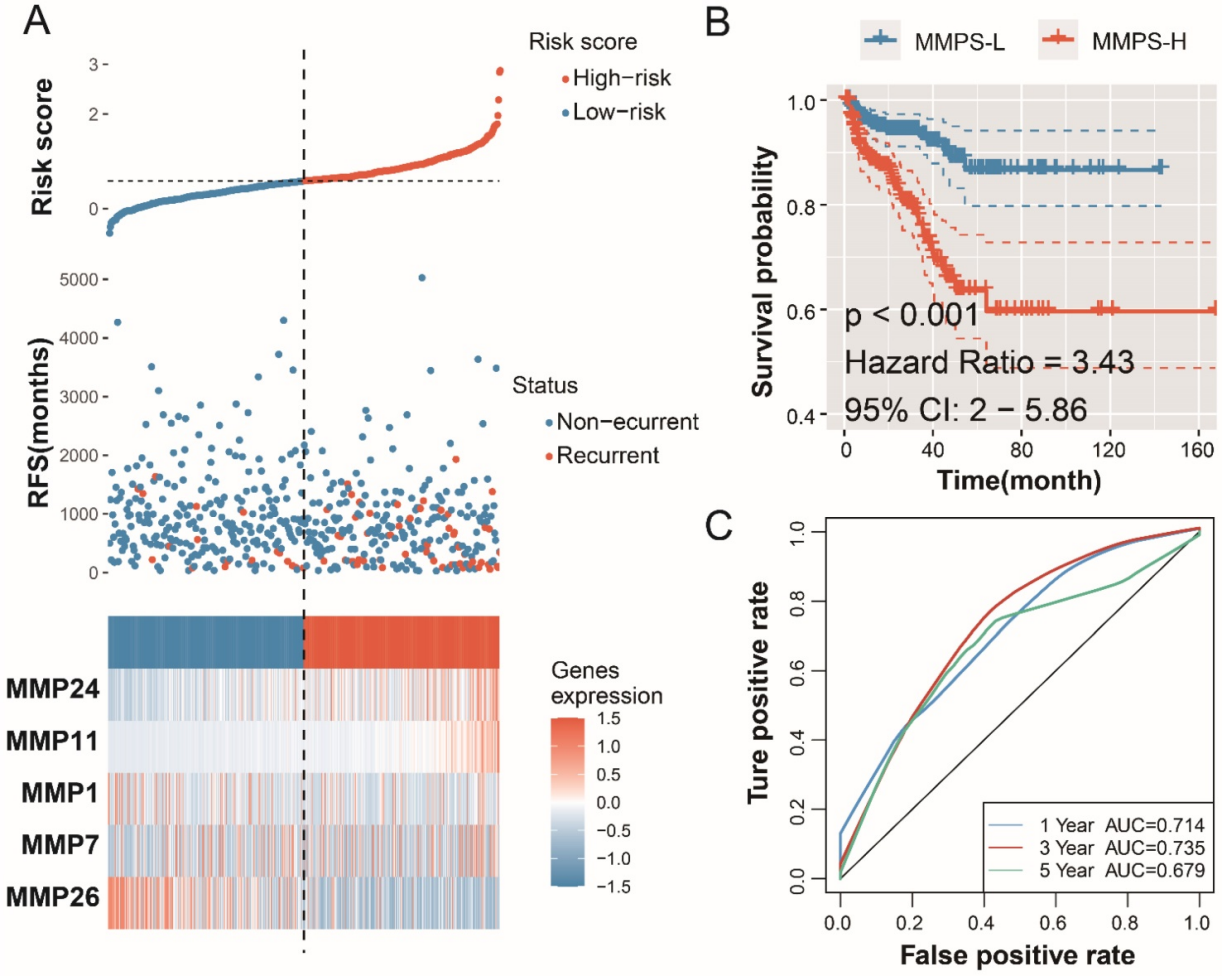
** Establishment of the MMP-related prognostic model in training TCGA-PRAD cohort.** (A) The risk score, recurrence status and five MMPs expression level; (B) K-M plot showed the RFS results of the MMPS-H (orange) and MMPS-L (blue) groups. (C) The 1-year, 3-year and 5-year ROC curves in the training group.

For each MMP, the LASSO analysis generated a coefficient, the risk score formula of the signature is: MMPS risk score= -0.04703*MMP1_exp_-0.0223* MMP7_exp_ +0.336547* MMP11_exp_ +0.218207* MMP24_exp_ -0.11865* MMP26_exp_. The risk score of each patient in TCGA-PRAD cohort, the outcome status of each patient and the expression of the enrolled five MMPs were displayed in Figure 4A. Patients with high risk, whose risk score higher than the median value of all the patients, are defined as the MMPS-H group, and the others are in MMPS-L group. Patients in MMPS-H group shown a poor prognosis and shorter RFS, as comparing with MMPS-L group (**Figure 4B**, HR=3.43, 95%CI=2-5.86, P<0.001). The predicted accuracy of the MMP-related signature is evaluated by the ROC curve, shown a good result with the 1-year (AUC=0.714), 3-year (AUC=0.735), and 5-year (AUC=0.679) RFS (**Figure 4C**).

### Validation of the MMP-related prognostic signature

**Figure 5:**
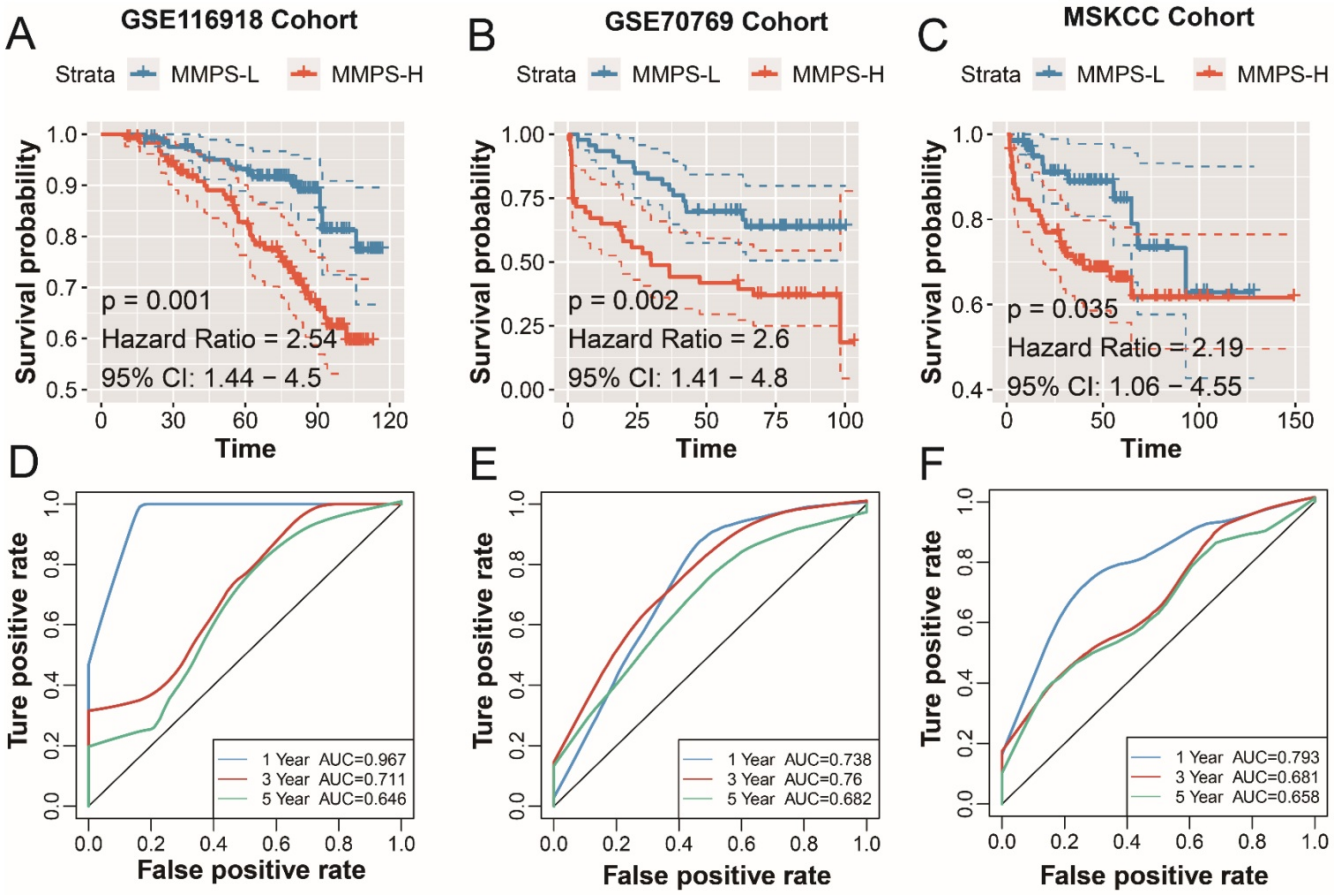
** Validation of the MMP-related prognostic model in external cohorts.** K-M plot showed the RFS results of the MMPS-H (orange) and MMPS-L (blue) groups in GSE116918 cohort (A), GSE70769 cohort (B) and MSKCC cohort (C); The 1-year, 3-year and 5-year ROC curves in GSE116918 cohort (D), GSE70769 cohort (E) and MSKCC cohort (F).

To confirm the prognostic value of the MMP-related signature in PCa patients. A total of 480 PCa patients from GSE116918, GSE70769 and MSKCC cohorts. The risk score of the patients was calculated with the above-mentioned formula. In GSE116918 cohort, patients in the MMPS-H group shown a poor prognosis with the HR of 2.54 (P=0.001,** Figure 5A**), the AUC of PCa recurrence at 1-year is high to 0.976, 0.711 for 3-year, and 0.646 for 5-year (**Figure 5D**). As to GSE70769, including 46 MMPS-H patients and 46 MMPS-L patients, the RFS time in MMPS-L group is longer (HR=2.6, 95%CI=1.41-4.8, P=0.002), and the prediction accuracy is reasonable (1-year AUC: 0.738; 3-year AUC=0.760, 5-year AUC=0.682, **Figure 5E**). The MMPS-H group in MSKCC cohort also displayed an unfavorable prognosis, and the HR value of the risk score is 2.19, with the 95% CI from 1.06 to 4.55 (P=0.035, **Figure 5C)**, and the short and long RFS prediction value is high than 0.65 (1-year AUC: 0.793; 3-year AUC=0.681, 5-year AUC=0.658, **Figure 5F**). To sum up, the MMP-related predict signature is with a high accuracy to judge the PCa recurrence.

### The MMPS is an independent risk for the prognosis of PCa patients

To evaluate the prognostic value of MMPS for PCa patients, we conducted the multivariate Cox analysis in each cohort with the clinical features (**Table 2**). In TCGA-PRAD cohort, Gleason score (HR: 2.43, 95%CI: 1.38-4.48, P=0.002), Stage (HR: 2.99, 95%CI: 1.30-6.86, P=0.009), and MMPS risk (HR: 1.92, 95%CI: 1.09-3.39, P=0.025) were the independent risk for the RFS outcome. The 248 patients in the GSE116918 cohort also illustrated the independent predict value of the MMPS (HR: 2.55, 95%CI: 1.05-6.19, P=0.039). As to GSE70769 cohort, Stage (HR: 3.09, 95%CI: 1.56-6.14, P=0.001), and MMPS risk (HR: 2.35, 95%CI: 1.22-4.51, P=0.010) acted as the independent risk for the RFS outcome. We failed to observe the independent predict value of MMPS (HR: 1.16, 95%CI: 0.58-2.34, P=0.67) after adjusting with the clinical features in MSKCC cohort.

### The potential MMPs driven mechanism to promote the progression of PCa

**Figure 6:**
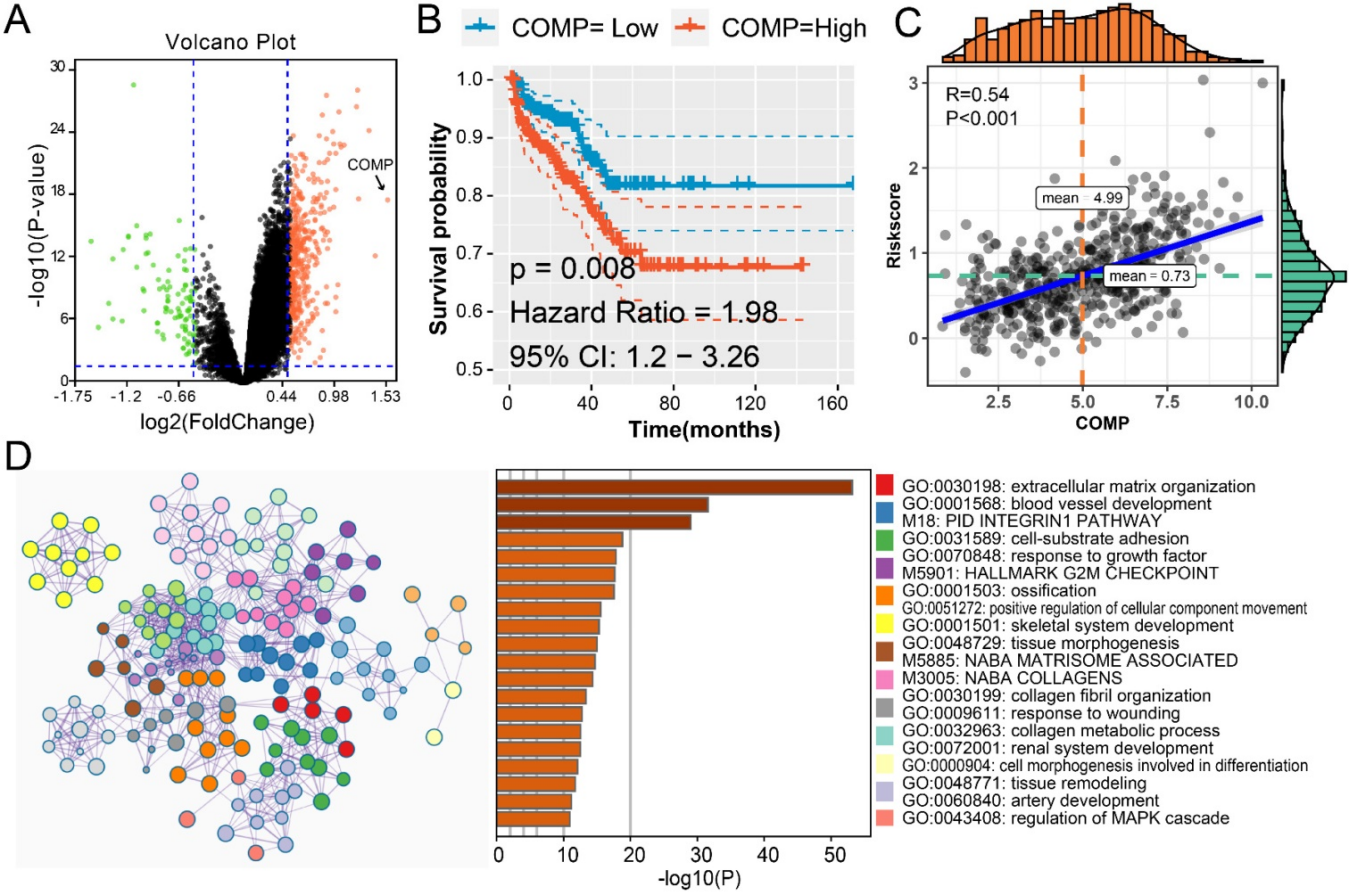
** The MMPs driven mechanisms in MMPS-H group.** (A) Volcano plot showed the DEGs among MMPS-H and MMPS-L groups; (B) K-M plot showed the RFS results of high and low mRNA level of COMP. (C) The correlation between COMP expression and MMPS risk score. (D) The pathway enrichment results of the 392 up-regulated genes in MMPS-H group.

To explore the possible mechanism caused by MMPs in the tumorigenesis of PCa, we firstly analyzed the DEGs in MMPS-H and MMP-L patients' groups. There are 394 up-regulated genes and 92 down-regulated genes with the cut-off fold change of 0.5 and P-value <0.05 (**Figure 6A**). COMP gene is the most elevated gene in MMPS-H group; with the help of K-M curve, we could see that the higher COMP, the higher risk of PCa recurrence (**Figure 6B**), and the association between COMP expression and risk score is high to 0.54 (**Figure 6C**). These results showed that the COMP is the potential MMPs driven downstream gene. We also use the Metascape to assess the gene enrichment of the upgrade of 394 genes. Not surprisingly, these genes are most enriched in GO:0030198: extracellular matrix organization, while the M18: Pid integrin1 pathway, M5901: HALLMARK G2M checkpoint and GO:0070848: response to growth factor were also illustrated, these pathways might be the target of PCa diagnosis and treatment.

### Immune infiltration of 22 type immunocytes

**Figure 7:**
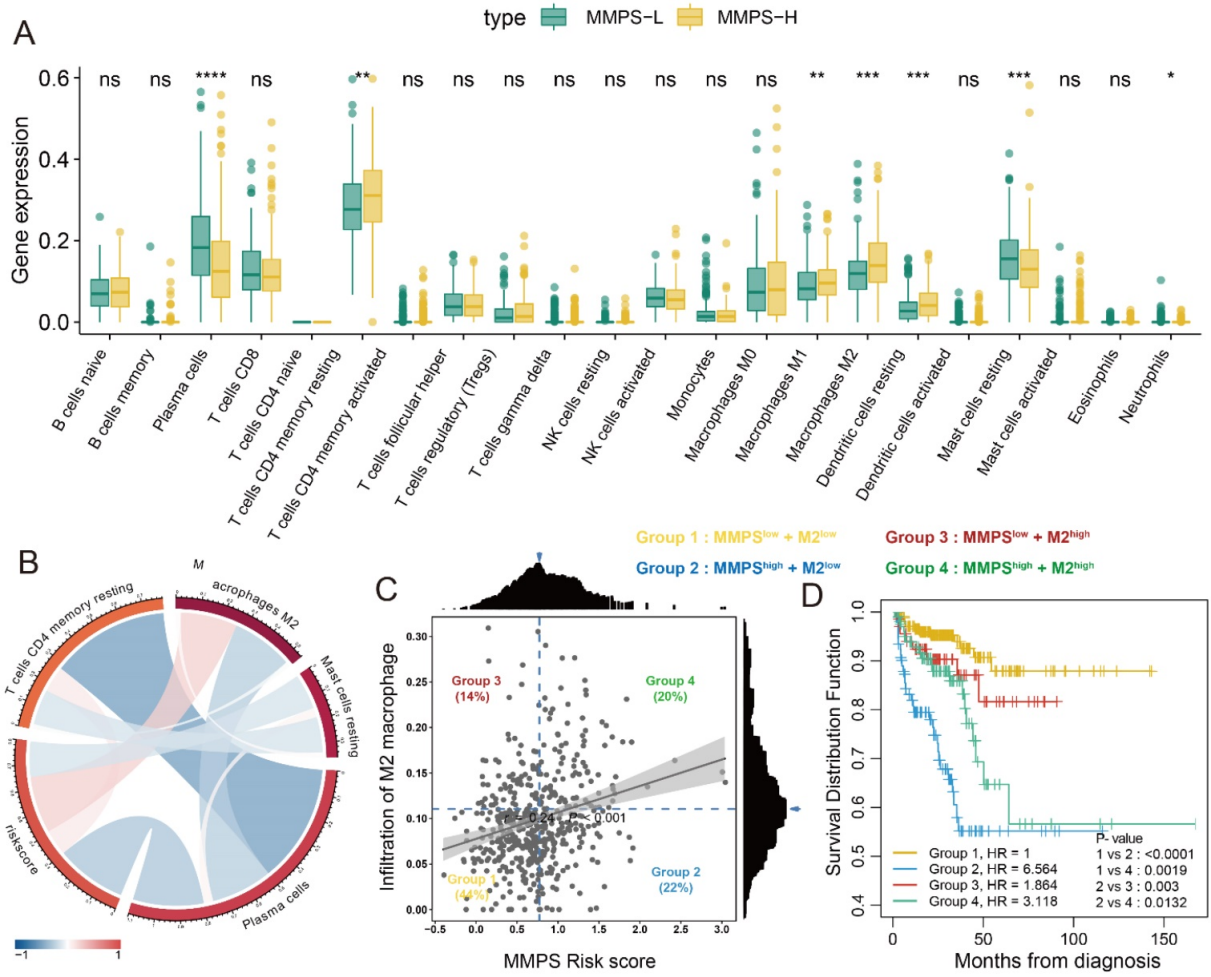
** Immune infiltration of 22 immunocytes and association with prognosis in PCa patients.** (A) Immune infiltration of 22 immunocytes in MMPS-L and MMPS-H groups; (B) The correlations between MMPS risk score and four immunocytes; (C) The optimal cut-off value of MMPS risk score and M2 macrophage infiltration to divide the enrolled patients to four groups; (D) K-M plot showed the different RFS of the four groups.

The results of the infiltration rate of 22 type immunocytes was generated by CIBERSORT. After comparing the infiltration of immunocytes of patients among MMPS-H and MMPS-L groups, we revealed that the plasma cells and resting mast cells decreased in MMPS-H group (all, P<0.05), while activated CD4+ memory T cells, M1, M2 macrophages and resting dendritic cells increased in MMPS-H group (all, P<0.05) (**Figure 7A**). We also found that the M2 macrophages are highly associated with the risk score generated by the MMP-related predict signature (R=0.24, **Figure 7B**). Meng *et al*. 26 recently reported that the M2 macrophage is a risk factor of PCa patients, Fakih *et al*. 27 reported a method to use the optimal cut-off to divide the enrolled patients to four groups by the scatter plot of two factors. Therefore, we use this method to separate the 496 TCGA-PRAD patients into four groups (**Figure 7C**). Interestingly, we found that the patients with low risk score all shown a better prognosis (Group I and III), no matter the infiltration of M2 macrophage is high or low, patients with the high MMPS risk score and low M2 macrophage showed the worst RFS (**Figure 7D**). These results showed that the MMPS score is an excellent signature to predict the prognosis.

## Discussion

Cancer invasion through dense extracellular matrices (ECMs) is mediated by MMPs, which degrade the ECM thereby creating paths for migration 28, 29. Mounting evidence has revealed the function of MMPs in the past years and MMPs are the pivotal mediators for the microenvironment alteration determined tumorigenesis 30, 31. The association between MMPs and PCa patients has also been widely studied. Białkowska *et al*. 32 reported that MMP7 rs11568818 polymorphism is correlated with the two-fold change of PCa risk, while MMP-1 rs1799750, MMP-2 rs243865, MMP13 rs2252070 not impact the risk. Ganguly *et al*. 33 found that Notch3 could promote the bone metastasis of PCa patients throng MMP3 mediated osteoblastic lesion formation. Kalantari *et al*. 34 revealed the bipartite function of MMP13 and TLR-9, patients with the high expression of MMP13 and TLR-9 showed an advanced stage of PCa. The status of CRPC and medicine resistance are the hot potatoes for the clinical treatment of PCa patients. Szarvas *et al*. 35 exposed higher pretreatment serum of MMP7 is the independent predictor of shorter cancer-specific survival and the resistance of docetaxel. Based on the above evidence, MMPs play an indispensable role in the initial alteration and development of PCa.

Due to the high recurrence rate and poor outcome of advanced PCa, and several researchers built the prognosis predict features to judge the outcome and provide more effeteness treatment for PCa patients. Toth *et al*. 36 generated a DNA methylation-based prognosis signature with the AUC of 0.95 in the training cohort, however, the AUC value in two external validation cohorts are only 0.771 and 0.687. Shao *et al*. 37 produced a seven long noncoding RNAs signature to predict the RFS of PCa, with the AUC value of 0.68 and C-index value of 0.63, whereas this study lacks external validation. Jiang *et al*. 38 developed a 15-gene signature using Elastic-net analysis, the signature showed a predict AUC value of 0.766 at 11.5 months, 0.738 at 22.3 months, and 0.764 at 48.4 months. Therefore, it is meaningful to establish the prognosis predict signature to distinguish the low risk and high-risk PCa patients, as well as provide the appropriate treatment for them.

In the current study, we comprehensively assess the expression and prognostic value of 22 MMPs in PCa patients. The mRNA level of MMPs in tumor and normal tissues is polarized, part of them increased in tumor tissues, part of them decreased in tumor tissues. What's more, about half of MMPs shown the negative relationship between the DNA methylation and mRNA expression, while genetic alteration is demonstrated no effect of mRNA level. Subsequently, the LASSO cox analysis was employed to dimensionality reduction and chose the MMPs to build the prognostic signature, MMP8, MMP21, MMP23B and MMP27 were excluded because of the lower expression of them. Finally, an MMP-related predict signature was obtained with the 1-year AUC of 0.714 in TCGA-PRAD training cohort and validated in three external cohorts with a high AUC value, including GSE116918, GSE70769 and MSKCC cohorts. What's more, after adjusting with the clinical features, we revealed that the MMPS signature is a robust independent predict toll for the RFS prognosis in PCa patients. The potential MMP-driven mechanisms also evaluated, and we reveled that Pid integrin1 pathway, G2M checkpoint and response to growth factor were the signaling pathways affected by MMPs. The positive associative between COMP and MMPS signature were also observed in this study. Liu *et al*. 39 reported that COMP is the biomarker for colon cancer and could promote the cell proliferation through Akt pathway. Stracke *et al*. 40 reported that MMP-19 may participate in the degradation of aggrecan and COMP in arthritic disease, whereas MMP-20 may primarily be involved in the turnover of these molecules during tooth development. Immunocyte infiltration was also concerned in this study, and found that the high risk score with the low infiltration of M2 macrophage shown the worst outcome in PCa patients. Based on the results generated from the current study, we could confirm the predict value of MMPS in PCa patients, in the future, if a patient diagnosed with the PCa and also obtained the high risk score of MMPS, we should take the active treatment to help him for the saving of the life.

## Conclusion

MMPs involved and played an essential role in the tumorigenesis and biochemical recurrence in PCa patients. The MMPS signature could accurately predict the recurrence of PCa patients, and validated in several cohorts. The MMPs could affect the progression of PCa through Pid integrin1 pathway, G2M checkpoint and response to growth factor pathways.

## Figures and Tables

**Table 1:** The information of training and validation cohorts

Cohorts	Total	Non-BCR (n)	BCR (n)	Download Link
TCGA-PRAD	496	426	90	https://gdc.xenahubs.net/download/TCGA-PRAD.htseq_fpkm.tsv.gz
MSKCC/GSE21032	140	105	35	http://cbio.mskcc.org/cancergenomics/prostate/data/
GSE116918	248	193	55	https://www.ncbi.nlm.nih.gov/geo/query/acc.cgi?acc=GSE116918
GSE70769	92	48	44	https://www.ncbi.nlm.nih.gov/geo/query/acc.cgi?acc=GSE70769

**Table 2:** Multivariate Cox analysis among MMPS signature and clinicopathological features.

Parameters	Recurrence-free Survival		
	HR	95% CI	P value
TCGA-PRAD			
Age (> 60 vs. ≤60)	1.14	(0.70-1.85)	0.595
Gleason score (≥8 vs. ≤7)	2.43	(1.38-4.48)	0.002*
Stage (≥T3 vs. ≤T2)	2.99	(1.30-6.86)	0.009*
Risk (MMPS-H vs. MMPS-L)	1.92	(1.09-3.39)	0.025*
GSE116918			
Age (> 60 vs. ≤60)	0.83	(0.38-1.8)	0.634
Gleason score (≥8 vs. ≤7)	0.53	(0.21-1.31)	0.169
Stage (≥T3 vs. ≤T2)	1.69	(0.93-3.05)	0.084
Risk (MMPS-H vs. MMPS-L)	2.55	(1.05-6.19)	0.039*
GSE70769			
Gleason score (≥8 vs. ≤7)	2.05	(0.99-4.22)	0.05
Stage (≥T3 vs. ≤T2)	3.09	(1.56-6.14)	0.001*
Risk (MMPS-H vs. MMPS-L)	2.35	(1.22-4.51)	0.010*
MSKCC			
Age (> 60 vs. ≤60)	0.80	(0.39-1.64)	0.543
Gleason score (≥8 vs. ≤7)	8.09	(3.79-17.24)	<0.001*
Stage (≥T3 vs. ≤T2)	2.97	(1.36-6.47)	0.006*
Risk (MMPS-H vs. MMPS-L)	1.16	(0.58-2.34)	0.67

*, P<0.05.
